# The outcomes and treatment strategies in metastatic soft tissue sarcoma treated with immunotherapy-based therapy: a three-center study

**DOI:** 10.3389/fimmu.2025.1504117

**Published:** 2025-03-28

**Authors:** Zhiyong Liu, Yao Weitao, Kang Cui, Songtao Gao, Xin Wang, Peng Zhang, Jiaqiang Wang

**Affiliations:** ^1^ Department of Bone and Soft Tissue Tumor, The Affiliated Cancer Hospital of Zhengzhou University and Henan Cancer Hospital, Zhengzhou, China; ^2^ Department of Oncology, First Affiliated Hospital of Zhengzhou University, Zhengzhou, China; ^3^ Department of Orthopedic, Henan Provincial People’s Hospital, Zhengzhou, China

**Keywords:** soft tissue sarcoma, immunotherapy, efficacy, adverse events, three-center

## Abstract

**Background:**

Preclinical studies showed that cytotoxic agents and antiangiogenic agents had regulatory effects in the tumor immune microenvironment of soft tissue sarcoma (STS), and then enhance the antitumor effect of immunotherapy. This study was to investigate the efficacy and safety of immunotherapy-based therapy in metastatic STS.

**Methods:**

We conducted a retrospective analysis in three centers where some patients received immunotherapy-based therapy consisting of immunotherapy alone or in combination with systemic agents (cytotoxic agents and/or antiangiogenic agents). The primary endpoints were median progression-free survival (mPFS) and median overall survival (mOS), and Kaplan-Meier method was used to compare survival.

**Results:**

A total of 79 patients were included in this study. With the median follow-up of 14.2 months, the mPFS and mOS was 7.5 months and 19.5 months, respectively. The PFS (P < 0.01) and OS (P < 0.01) were significantly better in the alveolar soft part sarcoma (ASPS) group compared to the non-ASPS group. Patients who treated in ≤2 lines had longer PFS (P < 0.01) and OS (P < 0.01) compared to those in subsequent lines. Further analysis was performed according to histopathological types, in patients with ASPS, the combination of immunotherapy-based therapy resulted in a longer PFS (P < 0.01) compared to immunotherapy in monotherapy. Similarly, the patients treated in ≤2 lines had longer PFS (P=0.03) and OS (P < 0.01) compared to in subsequent lines. In patients with non-ASPS, patients with potentially sensitive sarcomas (undifferentiated pleomorphic sarcoma, dedifferentiated liposarcoma, myxofibrosarcoma, and angiosarcoma) had a longer PFS (P = 0.02) and OS (P = 0.03) compared to other subtypes. The OS (P = 0.03) for patients with potentially sensitive sarcomas treated in ≤2 lines showed a long trend compared to subsequent lines. Most adverse events reported were mild and tolerable.

**Conclusions:**

The immunotherapy-based therapy showed promising activity in survival, especially in certain histological subtypes (undifferentiated pleomorphic sarcoma, dedifferentiated liposarcoma, myxofibrosarcoma, and angiosarcoma), as well as in combination treatment and in early lines. Prospective researches are needed to confirm the potential benefits.

## Background

Soft tissue sarcoma (STS) is a group of malignancy tumor of mesenchymal origin characterized by heterogeneities, accounting for approximately 1% of all adult malignancies, with approximately 39,900 new cases in China (1). STS has more than 70 different pathological subtypes with diverse clinical manifestations. The prognosis for advanced STS remains poor, with median progressive-free survival (mPFS) of about 4.2 months and median overall survival (mOS) of 15 to 18 months (2). Generally, chemotherapy with anthracyclines is considered the standard treatment for most advanced STS. Cytotoxic agents such as gemcitabine, eribulin, and ifosfamide can be selected for certain pathological subtypes in subsequent lines for more than three decades. New agents and treatment strategies have been introduced in China in recent years, antiangiogenic agents including pazopanib, anlotinib, and apatinib provide a new treatment option for advanced STS, especially in alveolar soft part sarcoma (ASPS) (3). However, those agents rarely lead to substantial survival benefits in metastatic or unresectable disease, with disease progression usually occurring after a modest mPFS of 2.5 months to 19 months. Therefore, novel agents and approaches are urgently needed to improve the prognosis.

Immunotherapy was first introduced in malignancies by injecting streptococcal organisms to stimulate the immune system. Modern immunological studies showed that patients with STS had the potential to benefit from immunotherapy due to a spectrum of immunogenicity, with varied levels of tumor-associated inflammation and immunogenicity (4). Immune checkpoint inhibitors (ICIs) demonstrated promising efficacy in several sarcoma subtypes. In the STS cohorts of SARC028 (5), pembrolizumab monotherapy obtained early promising effect with an overall response rate (ORR) of 20% for liposarcoma (LPS) and 40% for undifferentiated pleomorphic sarcoma (UPS). In the Alliance A091401 trial (6), ipilimumab and nivolumab yielded an ORR of 16% in patients with unselected STS, with the most tumor response occurring in myxofibrosarcoma (FBS), UPS, angiosarcoma (AS), and leiomyosarcoma (LMS). Atezolizumab monotherapy showed amazing efficacy in a phase II study including both adult and pediatric patients with advanced ASPS (7), with an ORR of 37% and mPFS of 20.8 months. However, the immunogenicity of the tumor microenvironment and the high interpatient heterogeneity contribute to sporadic therapeutic responses in STS, and preclinical studies demonstrated that cytotoxic and/or antiangiogenic agents could exhibit synergistic effects with immunotherapy by promoting tumor vascular normalization, tumor-associated macrophages, and neoantigen presentation through changes in the tumor microenvironment (8). To enhance the anti-tumor effect, the treatment strategies are shifting towards immunotherapy combined with conventional chemotherapy or anti-angiogenic therapy or other modalities. A phase II study including 37 patients with anthracycline-naive STS (9), showed that pembrolizumab and doxorubicin obtained preliminary promising benefits in unplanned subtypes, with an ORR of 36.7% and mPFS of 5.7 months. Two of 3 patients with UPS and 2 of 4 patients with DDLPS had durable partial response (PR). Another phase II study of pembrolizumab and eribulin for metastatic soft tissue sarcoma (mSTS) showed a 12-week progression-free survival rate (PFSR) of 36% for LMS (10), 69.6% for LPS, and 52.6% for UPS/others, respectively. These results suggested that immunotherapy-based therapy may provide a novel option for advanced STS.

The studies on immunotherapy for STS remains limited and the therapeutic effects are sometimes contradictory. More data is needed to fully understand the potential efficacy of immunotherapy-based therapy. Therefore, we analyzed the efficacy and toxicity of this strategy in a large cohort of patients receiving immunotherapy-based therapy at three treatment centers, and compared the efficacy of immunotherapy in monotherapy versus combination therapy, as well as the efficacy given in ≤2 lines versus subsequent lines. Additionally, we present two typical cases.

## Materials and methods

### Patients and treatment

This was a three-center retrospective study that included 52 patients with histologically confirmed mSTS at Henan Cancer Hospital from June 2019 to January 2024, 16 patients at the First Affiliated Hospital of Zhengzhou University from January 2022 to March 2023, and 11 patients at Henan Provincial People’s Hospital from January 2021 to March 2022. Patients who met the following criteria were included (1): aged 18 years or older; (2) had an Eastern Cooperative Oncology Group (ECOG) performance status of 0 or 3; (3) had at least one measurable lesion according to Response Evaluation Criteria in Solid Tumors, version 1.1 (RECIST 1.1), and the lesions would not be cured locally in subsequent therapies; (4) had not received previous immunotherapy, including anti-PD-1, anti-PD-L1, or anti-CTLA4. Patients who met the following criteria were excluded: (1) had only received one cycle of treatment; (2) had a history of immune disease or a second malignancy; (3) had no available follow-up data. In order to accurately assess the efficacy of immunotherapy for each subtype, only more than five patients with the same pathological subtype were included in this study.

Based on the medical records, we retrospectively analyzed the demographic characteristics, tumor characteristics, previous treatment details (surgery, systemic treatments), details of immunotherapy (therapeutic agents, number of systemic treatment lines), treatment course (start and end time of treatment, optimal response date, etc.), adverse events (AEs), and reasons for discontinuation. Due to the retrospective, anonymous, and non-interventional nature of the study, informed consent from all patients was waived and this study was approved by the Ethics Committee of each treatment center.

The formulations of treatment strategies were based on histopathological subtypes, previous treatment, available clinical trials, and patients’ willingness. The subtypes resistant to chemotherapy may be given immunotherapy in monotherapy or combination therapy as first-line treatment, for example, ASPS and clear cell sarcoma (CCS). While the subtypes sensitive to chemotherapy were rarely given immunotherapy as first-line treatment unless patients refuse or were not suitable for chemotherapy or anti-angiogenic therapy.

Immunotherapy, including sintilimab, camrelizumab, toripalimab, and geptanolimab, and anti-angiogenesis agents, such as apatinib, anlotnib, lenvatinib, and pazopanib, were included in the treatment regimens. Only cytotoxic agents recommended by National Comprehensive Cancer Network (NCCN) and Chinese Society of Clinical Oncology (CSCO) guidelines for advanced STS were given to patients, such as anthracyclines, ifosfamide, gemcitabine, and eribulin. These cytotoxic agents and anti-angiogenic agents were usually administered unless serious AEs, patient rejection, or disease progression occurred, or more than 6 cycles were completed. PD-1 inhibitors were given for a minimum of 2 and a maximum of 35 cycles. Combination therapy was defined as the PD-1 inhibitors combined with other systemic therapies, including chemotherapy and/or anti-angiogenic therapy. Patients who had to discontinue one drug due to serious toxicity were allowed to continue the other drug as monotherapy.

### Efficacy and safety evaluation

Imaging evaluations were usually performed every 2-3 months, and best responses at any point during the treatment are classified as complete response (CR) or PR, stable disease (SD), and progressive disease (PD) according to RECIST criteria. ORR referred to the proportion of patients with CR and PR, and disease control rate (DCR) referred to the proportion of patients with CR, PR, and SD. PFS was defined as the time from start of immunotherapy-based therapy to disease progression or death, and OS was defined as the time from start of immunotherapy-based therapy to death from any cause.

All patients were evaluated for safety, and AEs were collected and graded according to the Common Terminology Criteria for Adverse Events, version 5.0 (CTCAE 5.0). Usually, immunotherapy was continued until grade ≥ 3 AEs occurred, patients or clinicians refused the therapy, or disease progression was observed.

### Statistical analysis

For the descriptive analysis, the continuous variables were summarized with median, range, and numbers, and the categorical variables were summarized by frequency and percentage. The mPFS and mOS were the primary endpoints of this study. Survival was estimated and compared by the Kaplan–Meier method and the log-rank test for each group. The statistical significance was set at 0.05. Statistical analysis was carried out with Graphpad prism 8.0 (Graphpad Software, USA).

## Results

### Patient demography

In this study, 79 patients with mSTS were included. They were aged between 18 and 71, with the median age of 46.5. There were 42 males and 37 females. The most common primary sites were the trunk and extremities (89.9%), with the lung being the most common site of metastasis (72.2%). The most common pathological subtype was ASPS (n=20), followed by UPS (n=13), LMS (n=7), angiosarcoma (AS) (n=6), synovial sarcoma (SS) (n=6), FBS (n=6), CCS (n=6), dedifferentiated liposarcoma (DDLPS) (n=5), epithelioid sarcoma (ES) (n=5), and malignant peripheral nerve sheath tumor (MPNST) (n=5). The majority of patients had an ECOG performance status of ≤2. Treatment varied depending on the histopathological types. Out of the 79 patients, 72 (91.1%) had previously received systemic therapy, with the median of two lines (range 0-5). The majority (91.1%) of patients received combination therapy. Demographic characteristics was showed in **Table 1**.

**Table 1:** Patient Characteristics.

Variable	ASPS(n=20)	UPS(n=13)	DDLPS(n=5)	FBS(n=6)	AS(n=6)	SS(n=6)	LMS(n=7)	CCS(n=6)	MPNST (n=5)	ES(n=5)	Total(n=79)
Demographic and clinical characteristics											
Age (y)											
Mean/Median	24	56.8	46.7	56.7	64.6	46.7	51.4	38.5	45.2	43.2	46.5
Range	(18-35)	29-73	32-63	49-63	60-70	36-55	38-60	28-53	19-71	22-56	18-71
Sex(n)											
Female	9	6	3	3	3	2	3	2	3	3	37 (46.8%)
Male	11	7	2	3	3	4	4	4	2	2	42 (53.2%)
Primary site											
Extremities	16	9	3	3	3	3	4	5	3	4	53 (67.1%)
Retroperitoneum	0	1	1	1	0	0	0	0	0	0	3 (3.8%)
Trunk	4	3	1	1	1	1	3	1	2	1	18 (22.8%)
Others*	0	0	0	1	2	2	0	0	0	0	5 (6.3%)
Site of metastasis											
Lungs	16	9	5	4	3	5	5	4	3	3	57 (72.2%)
Others^#^	8	5	5	4	3	3	3	3	3	3	40 (50.6%)
Prior systemic therapies											
Median	1	2	3	2	2	3	2	1	2	1	2
Range	0-4	0-4	1-4	1-3	1-3	2-5	1-3	1-3	1-4	1-2	0-5
ECOG status											
0	10	8	4	3	4	4	4	5	4	3	49 (62.0%)
1	4	3	1	1	2	1	1	1	1	1	15 (20.0%)
2	4	1	0	1	0	1	1	0	0	1	9 (11.4%)
≥3	2	1	0	1	0	0	1	0	0	0	5 (6.3%)
Therapeutic regimens											
PD-1 inhibitors monotherapy	6	0	1	0	0	0	0	0	0	0	7 (8.9%)
PD-1 inhibitors +anti-angiogenesis	14	8	1	1	3	3	1	5	4	4	44 (55.7%)
PD-1 inhibitors+ chemotherapy	0	2	1	4	2	1	6	1	1	1	19 (24.1%)
PD-1 inhibitors + chemotherapy+ anti-angiogenesis	0	3	2	1	1	2	0	0	0	0	9 (11.3%)
Local treatment	4	3	1	1	1	1	1	1	0	0	13 (16.5%)

ECOG, Eastern Cooperative Oncology Group; ASPS, Alveolar soft part sarcoma; UPS, Undifferentiated pleomorphic sarcoma; DDLPS, Dedifferentiated liposarcoma; FBS, Myxofibrosarcoma; AS, Angiosarcoma; SS, Synovial sarcoma; LMS, Leiomyosarcoma; CCS, Clear cell sarcoma; MPNST, Malignant peripheral nerve sheath tumor; ES, Epithelioid sarcoma; Others*, Head and neck, mediastinum, etc; Others^#^, Bone, liver, etc.

Of 20 cases with ASPS, 6 received PD-1 inhibitor monotherapy, the others received a combination of PD-1 inhibitors and antiangiogenic agents. These combinations included anlotinib + sintilimab (n=6), apatinib + sintilimab (n=5), and anlotinib + camrelizumab (n=3). Out of these cases, 4 patients received local treatment for metastatic lesions during the therapy, with 2 undergoing radiotherapy and 2 undergoing radiofrequency ablation. While out of 59 non-ASPS cases, only one patient received PD-1 inhibitor monotherapy, 30 received a combination of PD-1 inhibitors and antiangiogenic agents, including anlotinib+ camrelizumab (n = 8), anlotinib+ sintilimab (n = 7), apatinib+ camrelizumab (n = 6), apatinib+ sintilimab (n = 5), and anlotinib+ triprolizumab (n = 4). 19 received a combination of PD-1 inhibitors and cytotoxic agents, with agents including doxorubicin-based (n = 4), gemcitabine-based (n = 8), ifosfamide-based (n = 4), and albumin paclitaxel-based (n = 3). 9 received a combination of PD-1 inhibitors, antiangiogenic agents, and cytotoxic agents, with agents including gemcitabine + anlotinib + sintilimab (n = 5), ifosfamide + apatinib+ camrelizumab (n = 2), and doxorubicin +anlotinib + sintilimab (n = 2). Local therapy was also given during the systemic treatment including radiotherapy (n = 3), radiofrequency ablation (n = 2), and surgery (n= 2).

### Efficacy

In the total population, there were 3 cases of CR, 15 cases of PR, 38 cases of SD, and 23 cases of PD. The ORR was 22.8% and the DCR was 70.9%. At the time of the current analysis, 7 patients were still on treatment, 72 discontinued. Of these patients, 66 (83.5%) discontinued due to disease progression, 3 (3.8%) due to AEs, and 3 (3.8%) for other reasons.

**Figure 1:**
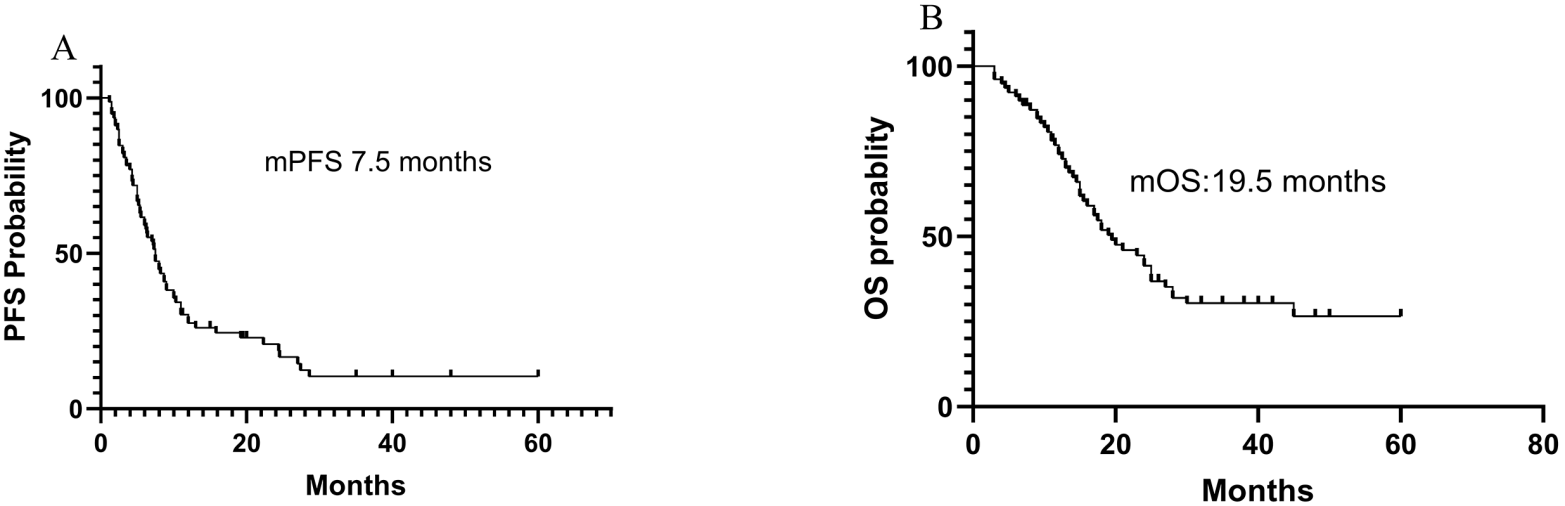
Efficacy of immunotherapy-based in total patients. **(A)** The PFS of all patients; **(B)** The OS of all patients.

The cutoff date was May 25, 2024, and the median follow-up period was 14.2 months. The mPFS and mOS was 7.5 months and 19.5 months, respectively. The PFS rates at 3 months, 6 months, and 12 months were 84.8%, 62.0%, and 27.8%, respectively. The OS rates at 6 months, 12 months, and 18 months were 89.8%, 70.9%, and 45.6%, respectively. **Figure 1** shows the efficacy in the total patients.

### Efficacy on ASPS, “potentially sensitive sarcomas”, and others

**Figure 2:**
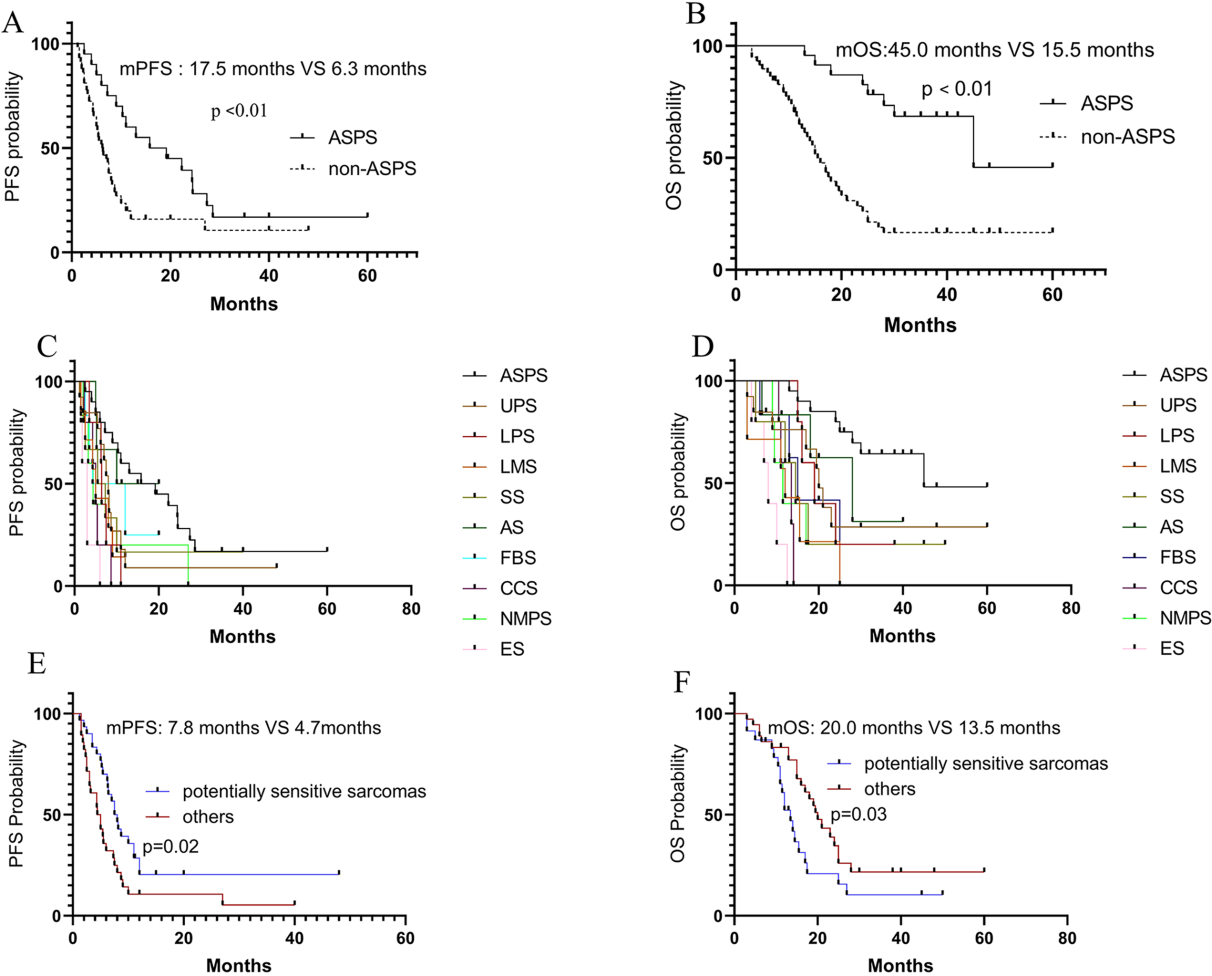
Efficacy in different subgroups. **(A, B)** PFS and OS in ASPS and non-ASPS; **(C, D)** PFS and OS in different sarcoma subtypes; **(E, F)** Comparison of PFS and OS between potentially sensitive sarcomas and others in non-ASPS.

Based on the sensitivity of sarcomas to immunotherapy, the subtypes were divided into ASPS and non-ASPS. In the ASPS group, 2 cases achieved CR and 8 achieved PR, resulting in an ORR of 50%. The mPFS and mOS was 17.5 months and 45 months, respectively. In the non-ASPS group, 1 case achieved CR and 7 achieved PR, resulting in an ORR of 13.6%. The mPFS and mOS was 6.3 months and 15.5 months, respectively. The efficacy in the two groups was shown in **Figures 2A, B**. It was observed that the PFS (*P*<0.01) and OS (*P*<0.01) in the ASPS group were longer than those in the non-ASPS group.

Further analysis was conducted on the efficacy in patients with different pathological subtypes, and the PFS and OS were shown in **Figures 2C, D**. According to previous studies and National Comprehensive Cancer Network (NCCN) guidelines (7, 11–15), we broadly divided STS into sensitive sarcomas (ASPS), potentially sensitive sarcomas (UPS, DDLPS, FBS, AS), and others (SS, ES, LMS, CCS, and MPNST). The results showed a trend of survival benefit for potentially sensitive sarcomas compared to others in terms of PFS (*P*=0.02) and OS (*P*=0.03) (**Figures 2E, F**).

### Difference in the efficacy of combination treatment and monotherapy

**Figure 3:**
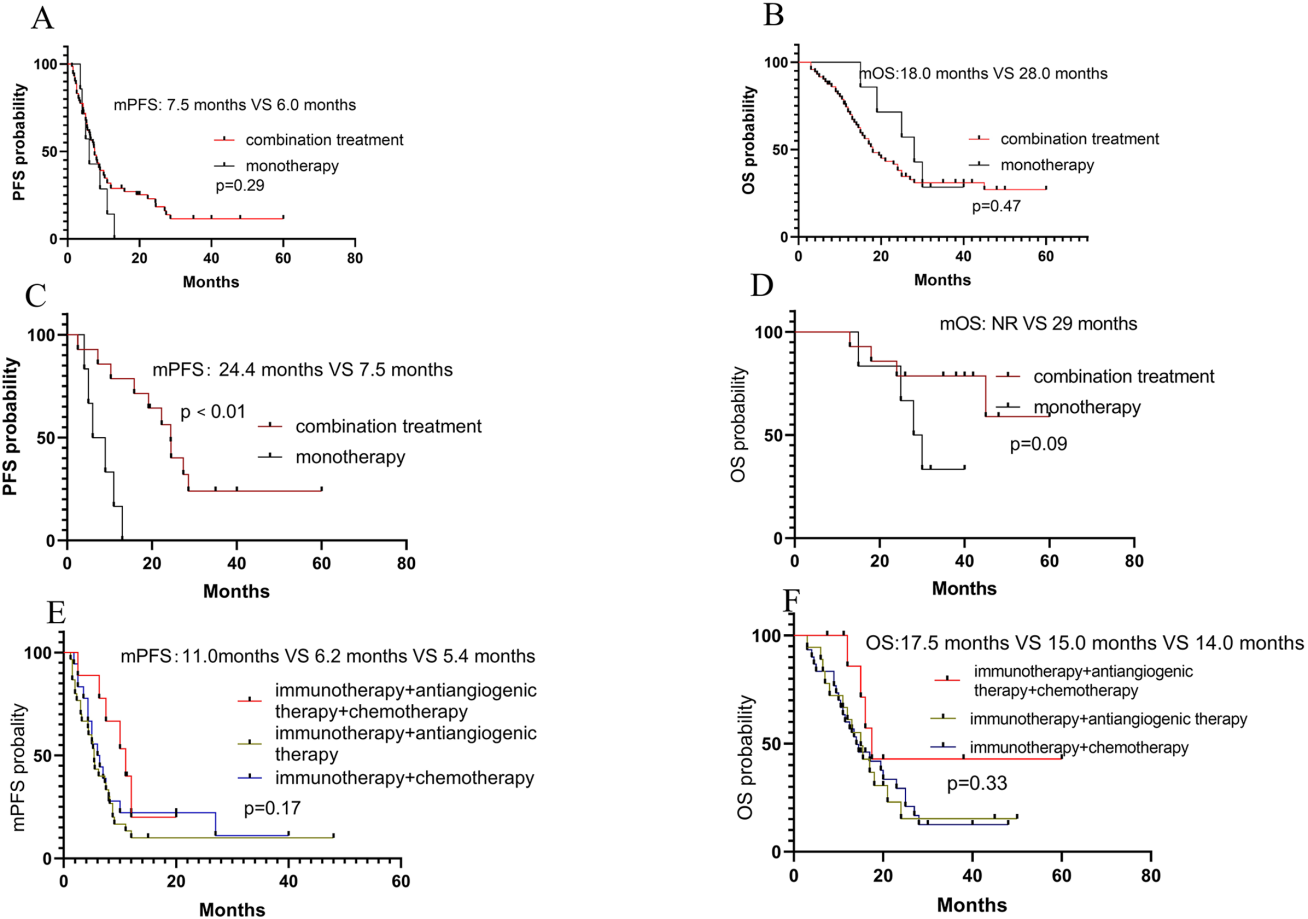
Survival analyses. **(A)** PFS and OS according to combination of therapy ((immunotherapy(I) alone versus combination in total patients); **(C, D)** PFS and OS according to combination of therapy (I alone versus combination in ASPS patients); **(E, F)** PFS and OS according to combination of therapy (I + anti-angiogenic agents (A) + cytotoxic agents (C) versus I+A versus I+C in non-ASPS patients.

Of the whole patients, 7 received PD-1 inhibitor monotherapy (6 with ASPS and 1 with non-ASPS), with the mPFS of 6 months and the mOS of 28 months, respectively. 72 (14 with ASPS and 58 with non-ASPS) received PD-1 inhibitors and other systemic agents, with the mPFS of 6 months and the mOS of 18 months, respectively. No significant differences in terms of PFS (*P*=0.29) and OS (*P*=0.47) were observed between the two treatment modalities (**Figures 3A, B**).

Among the 20 patients with ASPS, 14 received PD-1 inhibitors and antiangiogenic agents, with the mPFS of 24.4 months and the mOS of not reached (NR), respectively. While the others received PD-1 inhibitors monotherapy, with the mPFS of 7.5 months and mOS of 29 months, respectively. A significant difference in PFS (*P*<0.01) was observed between the two treatment modalities, but no significant difference in OS (*P*=0.09) (**Figures 3C, D**). Among the non-ASPS patients, 19 patients received a combination of PD-1 inhibitors and cytotoxic agents, with the mPFS of 6.2 months and the mOS of 15.0 months, respectively, while 30 received a combination of PD-1 inhibitors and antiangiogenic agents, with the mPFS of 5.4 months and the mOS of 14 months, respectively. 9 received a combination of PD-1 inhibitors, cytotoxic agents, and antiangiogenic agents, with the mPFS of 11 months and the mOS of 17.5 months, respectively. These results suggested that the combination of PD-1 inhibitors, cytotoxic agents, and antiangiogenic agents may lead to a great prognosis in terms of PFS and OS (**Figures 3E, F**). Additionally, 8 patients received local treatment, including radiotherapy and ablation.

### Difference in efficacy between ≤2 lines and sequent lines

**Figure 4:**
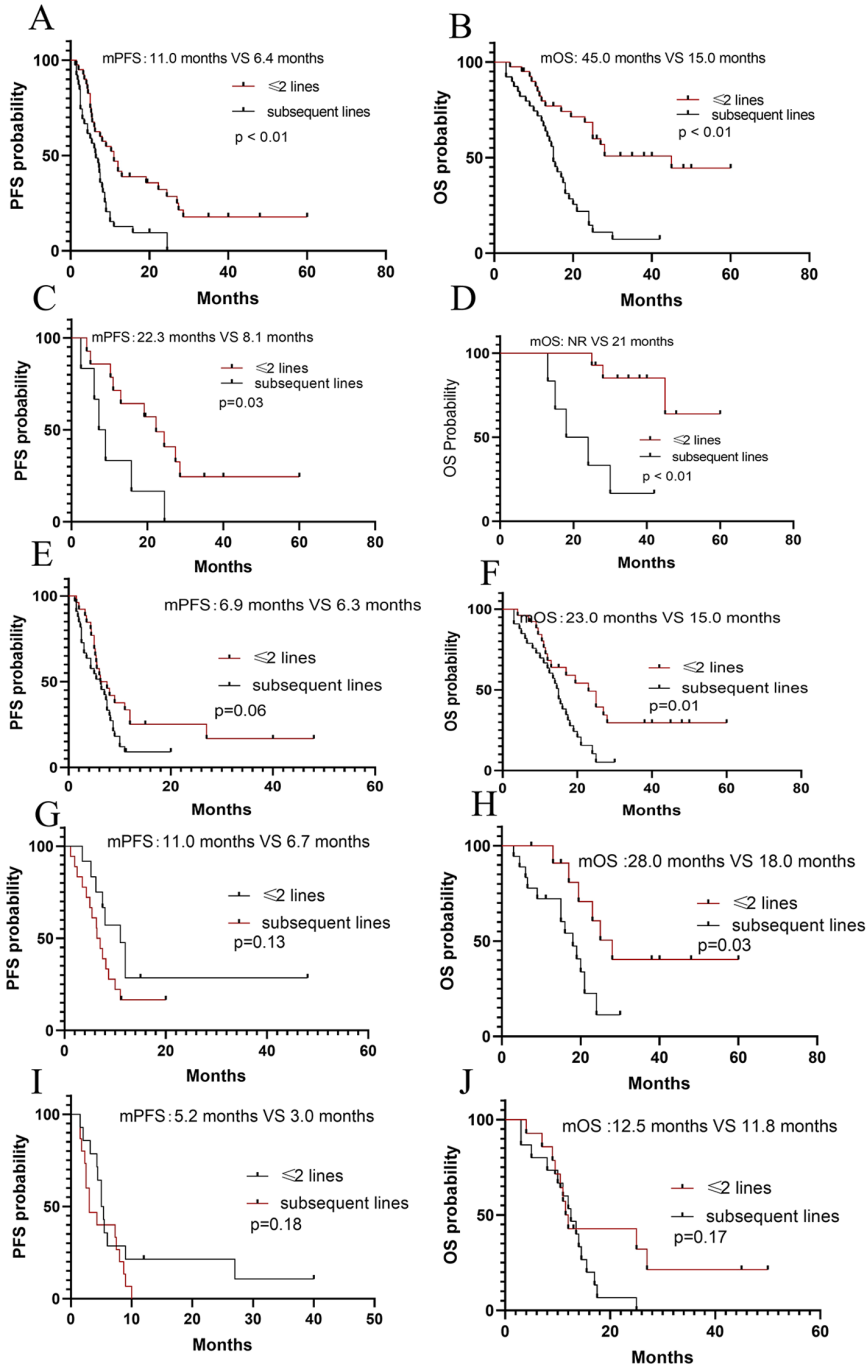
Survival analyses. **(A, B)** PFS and OS according to line of therapy (treatment in ≤2 lines versus subsequent lines in total patients); **(C, D)** PFS and OS according to line of therapy (treatment in ≤2 lines versus subsequent lines in ASPS patients); **(E, F)** PFS and OS according to line of therapy (treatment in ≤2 lines versus subsequent lines in non-ASPS patients); **(G, H)** PFS and OS according to line of therapy (treatment in ≤2 lines versus subsequent lines in potential sensitive sarcomas); **(I, J)** PFS according to line of therapy (treatment in ≤2 lines versus subsequent lines in other sarcomas).

Totally, there were 37 patients (14 patients with ASPS and 23 patients with non-ASPS) who were treated in ≤2 lines, with the mPFS of 11 months and the mOS of 45 months, respectively. In subsequent lines, there were 6 patients with ASPS and 36 with non-ASPS patients, with the mPFS of 6.4 months and the mOS of 15 months, respectively. Both the PFS (*P* < 0.01) and OS (*P*<0.01) of the patients treated in ≤2 lines were longer compared to those treated in subsequent lines (**Figures 4A, B**).

Subgroup analysis, among 20 patients with ASPS, 14 were treated in ≤2 lines with the mPFS of 22.3 months and the mOS of NR, respectively. While 6 ASPS patients were treated in subsequent lines with the mPFS of 8.1 months and the mOS of 21 months, respectively. The results showed a trend of survival benefit for patients treated in ≤2 lines compared to in subsequent lines in terms of PFS (*P*=0.03) and OS (*P* < 0.01). (**Figures 4C, D**). An analysis of the efficacy in ≤2 lines and subsequent lines in non-ASPS patients was also conducted. 25 non-ASPS patients were treated in ≤2 lines with the mPFS of 6.9 months and the mOS of 23 months, respectively. While 33 non-ASPS patients were treated in subsequent lines with the mPFS of 6.3 months and the mOS of 15 months, respectively. A trend of survival benefit for non-ASPS patients treated in ≤2 lines compared to in subsequent lines in terms of OS (*P*=0.01), and no statistical significance were observed in PFS (*P*=0.06) (**Figures 4E, F**). Additionally, it was found that patients with potentially sensitive sarcomas had a trend of benefit in OS in ≤2 lines (*P*=0.03) compared to in subsequent lines, but no significant benefit was observed in PFS (*P*=0.13) (**Figures 4G, H**). No significant difference in either PFS (*P*=0.18) or OS (*P*=0.17) between patients with other types treated in ≤2 lines and subsequent lines (**Figures 4I, J**).

### AEs

In the entire population, the majority of AEs related to treatment were well-tolerated and manageable, consistent with previous reports. Out of the 51 patients included in this study, 63 (79.7%) experienced one or more AEs. The most commonly AEs of any grade were fatigue (n = 46, 58.2%), gastrointestinal reactions (n = 44, 55.7%), myelosuppression (n = 29, 36.7%), renal inadequacy (n = 22, 27.8%). Due to the retrospective nature of the study, grade 1-2 AEs may not be accurately recorded. The most common grade 3-5 AEs were myelosuppression (n = 14, 17.7%), gastrointestinal reactions (n = 5, 6.3%), hypertension (n = 2, 2.5%). Grade 3-4 AEs related to immunotherapy included cytokine release syndrome (n=1), hyperglycemia (n=1). No grade 5 AEs related to the treatment were observed, and 6 (8.6%) patients discontinued treatment permanently due to the toxicities of PD-1 inhibitors or patient rejection. More details of toxicity are depicted in **Table 2**.

**Table 2:** Adverse events.

Variable	Grade I	Grade II	Grade III	Grade IV	Total
Adverse events					
Myelosuppression	6(7.6%)	9(11.4%)	8(10.1%)	6(7.6%)	29(36.7%)
Gastrointestinal reactions	25(31.6%)	14(17.7%)	5(6.3%)	0	44(55.7%)
Fatigue	31(39.2%)	15(19.0%)	0	0	46(58.2%)
Abnormal liver function	8(10.1%)	7(8.9%)	0	0	15(19.0%)
Renal inadequacy	15(19.0%)	6(7.6%)	1(1.3%)	0	22(27.8%)
Hypertension	7(8.9%)	4(5.1%)	2(2.5%)	0	12(16.5%)
Thyroid dysfunction	6(7.6%)	3(3.8%)	0	0	9(11.4%)
Rashes and other skin adverse reactions	4(5.1%)	2(2.5%)	0	0	6(7.6%)
Hyperglycemia	0	1	0	1(1.3%)	2(1.5%)
Cytokine release syndrome	0	0	0	1(1.3%)	1(1.3%)

### Some typical cases of immunotherapy-based therapy

**Figure 5:**
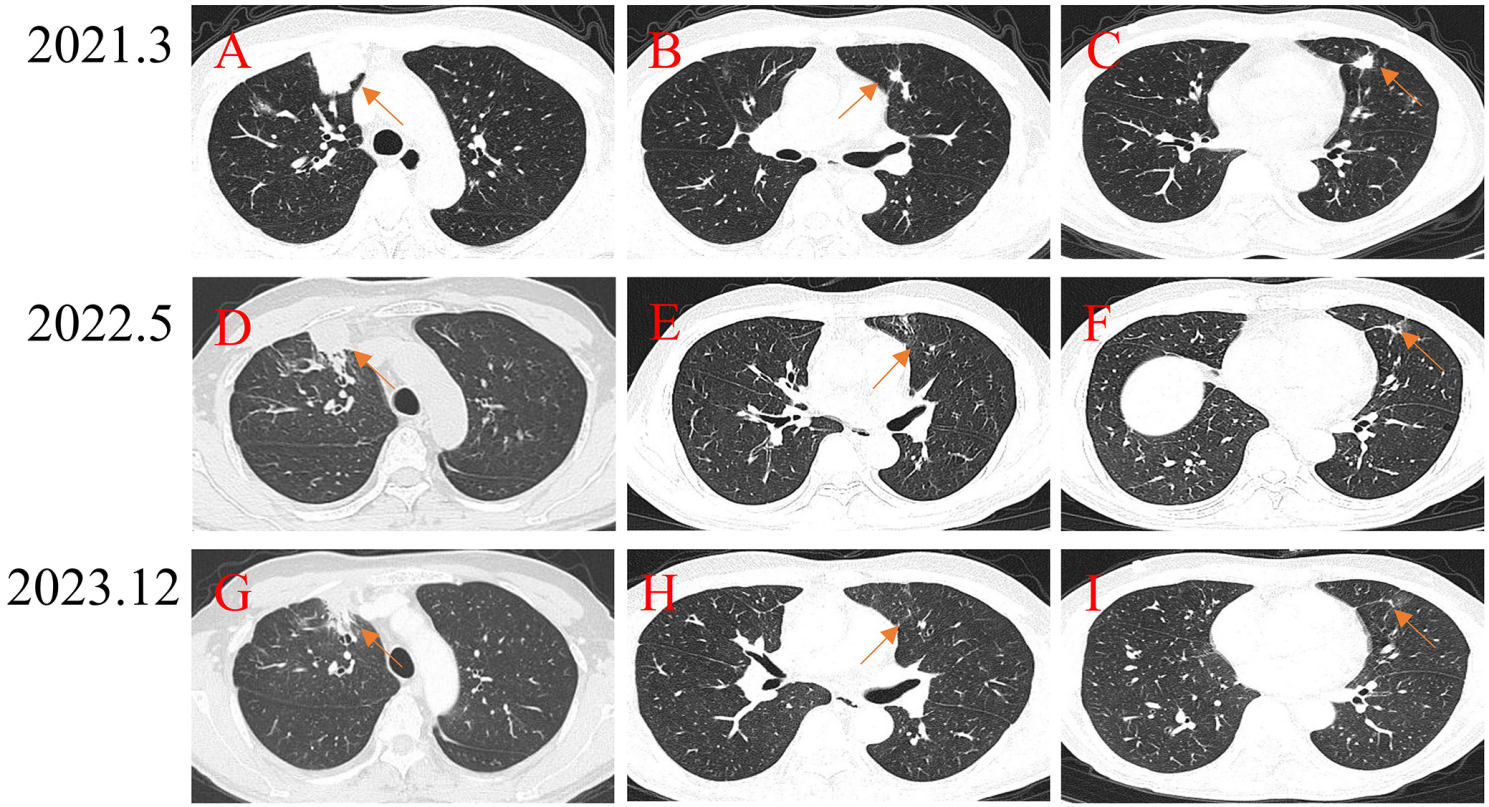
Treatment of a patient with high-grade UPS of the right thigh with lung metastases using PD-1 inhibitor + chemotherapy. **(A-C)** Pretreatment CT scan shows multiple lung metastatic lesions. **(D-F)** After receiving PD-1 inhibitor-based combination therapy, the tumor continuously shrinks. **(G-I)** Seventeen months after stopping PD-1 inhibitor-based combination therapy, the tumors continued to shrink.

A patient with high-grade UPS of the right thigh experienced recurrence 1 year after surgery, accompanied by multiple lung metastatic lesions. To control the lung lesions, the patients received a sequence of doxorubicin and ifosfamide, anlotinib, and gemcitabine, however the lung lesions continued to progress (**Figures 5A-C**). To activate the immune response, the patient received apatinib and carrilizumab. After 15 months of the combination treatment, the metastatic lesions shrinked (**Figures 5D-F**) and the patient stopped the combination treatment due to anorexia. Until data analysis, the lesions have remained stable for 19 months after stopping all drug therapy (**Figures 5G-I**).

**Figure 6:**
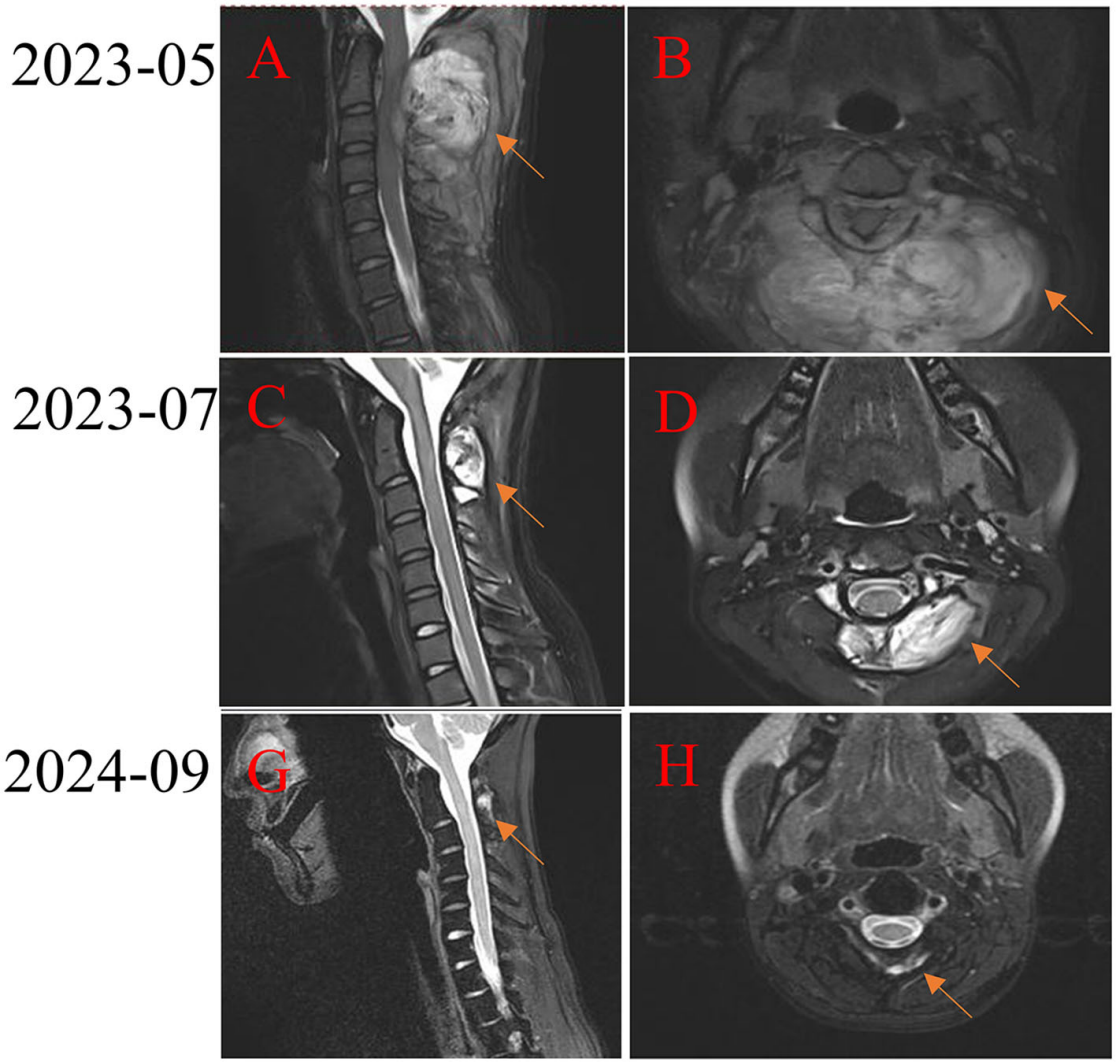
Treatment of a patient with high-grade malignant peripheral nerve sheath tumor of cervical spinal with PD-1 inhibitor. **(A, B)** Pretreatment MRI scan shows a large tumor in the cervical spinal. **(C-H)** After receiving PD-1 inhibitor-based combination therapy, the tumor continuously shrinks.

A patient with a malignant peripheral nerve sheath tumor of cervical spinal with a maximum diameter of 9.4 cm accompanied by multifocal lung metastasis (**Figures 6A, B**). Doxorubicin, ifosfamide, and toripalimab was given in the first-line treatment. MRI examination showed a significant reduction in lung metastasis after 1 cycle of toripalimab combined with chemotherapy (**Figures 6C, D**). Despite stopping chemotherapy after six cycles, the tumors continued to shrink during maintenance therapy with toripalimab (**Figures 6E, F**).

## Discussion

The main treatment options for advanced STS are chemotherapy and anti-angiogenic therapy. However, due to the heterogeneous subtypes and limited treatments, the survival of patients with advanced STS remains a bottleneck period. Therefore, new strategies are urgently needed to improve the survival. With the promising effects of immunotherapy in some subtypes, the efficacy and safety of immunotherapy-based therapy in advanced STS have been explored by a growing number of studies. We believe that our study was one of the largest studies to evaluate the efficacy and safety of immunotherapy-based therapy in advanced setting in the Eastern population. The immunotherapy-based therapy showed clinically meaningful activity and good tolerance, with the mPFS of 7.5 months and the mOS of 19.5 months, respectively. Further analysis revealed that immunotherapy-based therapy may benefit patients with certain histological subtypes (ASPS, UPS, DDLPS, FBS, AS), those who received combination therapy, and those who were treated in earlier treatment lines. However, the potential benefits need to be confirmed by further prospective studies.

In this study, 59 (74.7%) patients were diagnosed with non-ASPS, and 70(88.6%) received previously systemic therapy. 72(91.1%) received combination therapy, and 50(63.3%) received immunotherapy in second or subsequent lines. Similar characteristics at baseline were observed in several phase II studies, suggesting that immunotherapy may be a viable option, especially given in combination and in later lines, and in potentially sensitive sarcomas (UPS, DDLPS, FBS, and AS). These subtypes are characterized by a high level of genomic instability, as indicated by their complex karyotype with high copy number alterations, and these features could lead to a high potential for immunogenicity through increased neoantigen formation, and then play a role in determining the response to immunotherapy. Historical data of immunotherapy-based therapy showed that the mPFS for advanced ASPS was 6.2 months to 23.06 months, which was significantly higher than the mPFS of 4.2 to 8 months for other subtypes (16). Similarly, our study showed patients with potentially sensitive sarcomas had a trend of survival advantage over others in non-ASPS. The advantages of immunotherapy may be the enduring benefits, even after the treatment is discontinued, similar to those in squamous cell cancer and melanoma. Although this benefit has rarely been reported, we observed a UPS patient with multiple lung metastases which remained stable for 19 months after discontinuing all medications, as illustrated by the typical case above. Unfortunately, we didn’t have a molecular explanation for this observation. Additionally, our findings suggested that immunotherapy given in ≤ 2 lines resulted in better PFS and OS compared to subsequent lines. Similar results were observed in a meta-analysis, which demonstrated the importance of early initiation of immunotherapy to improve OS in patients with advanced non-small cell lung cancer (17). Regarding the treatment strategies, our studies showed that the combination treatment obtained the greater PFS and/or OS compared to the immunotherapy in monotherapy in advanced setting, especially in ASPS. which could be interpreted as the change of the tumor microenvironment, resulting in a synergistic effect.

The chemotherapy-resistant ASPS cohort showed notable improvements in survival with the introduction of antiangiogenic therapy. Antiangiogenic agents such as pazopanib, cediranib, sunitinib, and anlotinib have demonstrated significantly improved PFS and OS in patients with metastatic ASPS, reaffirming the potential of antiangiogenic agents in treating this malignancy. Historical data showed that the rate of 5-year OS was about 22% in 1989, which increased to about 61% in 2018 in the metastatic setting (18). Currently, antiangiogenic agents are considered the preferred and most effective treatment for advanced ASPS. Importantly, there is no evidence of complete cross-resistance between different antiangiogenic agents. Despite ASPS having a low tumor mutation load and microsatellite stability, it could still benefit from immunotherapy in monotherapy. A notable study of 52 patients with advanced ASPS showed that atezolizumab achieved an ORR of 37% and the mPFS of 20.8 months (7), with the median duration of response of about 25 months. In our present study, we included 20 cases of metastatic ASPS treated with immunotherapy-based therapy, with the mPFS of 17.5 months and the mOS of 45 months, respectively, which were comparable to or slightly better than previous studies. Further analysis showed that the immunotherapy combination treatment resulted in a significantly better PFS than monotherapy. However, the benefit did not translate into the advantage of OS. Additionally, patients receiving immunotherapy-based therapy in ≤2 lines had a longer PFS compared to those in subsequent lines. A retrospective worldwide registry of 76 patients with metastatic ASPS reported an ORR of 54.4% and mPFS of 16.3 months (16), patients who received immunotherapy as a first-line treatment achieved greater PFS than subsequent lines (NR *vs* 12.1 months). One possible mechanism for the acquired resistance to immunotherapy was the activation of immune evasion by macrophages, which may be caused by changes in the immune microenvironment due to antiangiogenic agents (19). This observation was also found in patients with advanced melanoma who experienced only limited benefit after treatment failure with BRAF/MEK inhibitors.

In the characteristic of “cold tumors” in non-ASPS, ICIs monotherapy showed disappointing antitumor activity, except for pembrolizumab in SARC028. Combination strategies involving cytotoxic agents and/or antiangiogenic agents have been commonly used to convert these tumors into “hot tumors” and increase their sensitivity to ICIs (2). A recent data analysis from nine clinical trials showed that PD-1/PD-L1 monoclonal antibodies only achieved an ORR of 9.8% in non-ASPS (20). Therefore, current clinical trials focused on immunotherapy as a combination therapy or for specific subtypes. For example, a phase II trial involving patients with advanced anthracycline-naive sarcoma treated with pembrolizumab plus doxorubicin showed promising effects (21), with the mPFS of 8.1 months and the mOS of 27 months in unplanned subtypes. Pembrolizumab plus olaratumab demonstrated an ORR of 21.4% in patients with advanced STS in a Phase I a/b study (22). The SAINT study showed that ipilimumab, nivolumab, and trabectedin as first-line treatment for advanced STS obtained the mPFS of 6.7 months and the mOS of 24.6 months (23), with an ORR of 25.3% and a DCR of 87.3%. Our study also showed modest efficacy, with the mPFS of 6.3 months and the mOS of 15.5 months, respectively. Although cross-trial comparisons should be interpreted with caution, the efficacy of our study was still notable, which may be attributed to the high proportion of multimodal combination therapy and patients with potentially sensitive sarcoma. A randomized phase II study compared the efficacy between cabozantinib alone with a combination of nivolumab, ipilimumab and cabozantinib in mSTS (24). The results showed that the PFS for the combination therapy was significantly longer than that for the monotherapy (5.3 months *vs* 3.5 months). It is generally known that certain subtypes, such as UPS, AS, DDLPS, and FBS, may be sensitive to immunotherapy in monotherapy. The present study showed that these potentially sensitive subtypes had a trend toward improved PFS and OS. A phase II study of 38 patients with advanced STS treated with sintilimab and doxorubicin showed that UPS and DDLPS had significantly longer PFS than other subtypes (25). Therefore, it is reasonable to consider immunotherapy-based therapy as a potential option for potentially sensitive sarcomas, especially for patients with limited options. Our study also explored rational combination strategies and found that multimodal therapy, especially the combination of PD-1 inhibitors, cytotoxic agents, and antiangiogenic agents, had promising activity, which was consistent with a previous study published by Zhou et al, whose report showed the combination of penpulimab, anlotinib, and eribulin as first-line treatment for 32 patients with advanced STS achieved impressive PFS of 10.55 months and an ORR of 12.50% (26). Additionally, no significant differences in efficacy were observed between immunotherapy combined with cytotoxic agents and immunotherapy combined with antiangiogenic agents, which suggested that the combination of antiangiogenic agents and immunotherapy may be a preferred option for patients who were unable to tolerate the toxicities of chemotherapy. In terms of treatment lines, PFS and OS for patients treated in ≤ 2 lines had long trend compared to those given in subsequent lines in non-ASPS patients, and patients were treated in ≤ 2 lines showed a benefit of OS compared to treated in subsequent lines in potentially sensitive sarcoma, which similar to in advanced head and neck squamous cell carcinoma (27), pembrolizumab combined with platinum and 5-fluorouracil improved OS, but not PFS, compared with cetuximab combined with chemotherapy. However, similar results were not observed in other subtypes in non-ASPS.

The toxicity profile in this study was similar to previous observations. Most of the AEs were mild and manageable. The majority of Grade ≥3 AEs were myelosuppression. It was worth noting that some of the toxicities related to immunotherapy observed in this study were emergencies, such as cytokine storms and diabetic ketoacidosis caused by hyperglycemia. While there is a lack of consensus on detections of AEs related to immunotherapy, it is important to regularly monitor indicators of toxicity and promptly address any toxicities.

This study has several limitations. Firstly, although a big number of patients were included in the study, there were still some highly heterogeneous indicators, such as previous treatment, combination treatment strategies, and patient and tumor characteristics. Therefore, future prospective studies with larger sample sizes and more homogeneous cohorts are warranted to obtain a comprehensive understanding of immunotherapy-based therapy. Secondly, it was important to note that in the respective study, patient treatment strategies and agents employed could vary due to many factors. Although our study identified an important role of immunotherapy in the treatment of STS, further clinical researches are needed to determine the optimal treatment strategy and agents. Thirdly, the generalizability of our study may be limited as the patients in our centers were mainly from central China. However, by 2024, the population of the central region is expected to exceed 100 million. As departments of three large university-affiliated tertiary general hospitals, the included centers had extensive experience in the management of advanced STS in the region. This could potentially enhance the applicability of our findings to a larger population. Fourthly, this study only analyzed the main factors that may affect the prognosis, including pathological subtypes, treatment regimens and the number of treatment lines. Other factors that may influence prognosis, including age and sex, were not analyzed.

## Conclusion

In conclusion, our study showed immunotherapy-based therapy showed promising efficacy in patients with mSTS, especially with ASPS, UPS, DDLPS, FBS, and AS, as well as in combination treatment and given in early lines. The majority of AEs were mild and manageable. Further research is needed, including recruiting more patients and conducting well-designed prospective studies, to confirm the efficacy of these treatment strategies in advanced STS.

## Data Availability

The original contributions presented in the study are included in the article/supplementary files, further inquiries can be directed to the corresponding author/s.

## References

[1] Liao Z Teng J Li T Liu H Li T Zhang C. Evaluation of the efficacy and safety of immunotherapy in sarcoma: a two-center study. Front Immunol. (2024) 15:1292325. doi: 10.3389/fimmu.2024.1292325 38585276 PMC10995229

[2] Spalato-Ceruso M Ghazzi NE Italiano A. New strategies in soft tissue sarcoma treatment. J Hematol Oncol. (2024) 17:76. doi: 10.1186/s13045-024-01580-3 39218932 PMC11368005

[3] Haddox CL Baldini EH Jagannathan JP Hornick JL Raut CP. Multidisciplinary approach for a high-risk, localized soft tissue sarcoma of the trunk after unplanned nononcological resection. CA Cancer J Clin. (2023) 73:451–7. doi: 10.3322/caac.21787 37226418

[4] Pollack SM He Q Yearley JH Emerson R Vignali M Zhang Y. T-cell infiltration and clonality correlate with programmed cell death protein 1 and programmed death-ligand 1 expression in patients with soft tissue sarcomas. Cancer. (2017) 123:3291–304. doi: 10.1002/cncr.30726 PMC5568958 28463396

[5] Keung EZ Burgess M Salazar R Parra ER Rodrigues-Canales J Bolejack V. Correlative analyses of the SARC028 trial reveal an association between sarcoma-associated immune infiltrate and response to pembrolizumab. Clin Cancer Res. (2020) 26:1258–66. doi: 10.1158/1078-0432 PMC7731262 31900276

[6] D’Angelo SP Mahoney MR Van Tine BA Atkins J Milhem MM Jahagirdar BN. Nivolumab with or without ipilimumab treatment for metastatic sarcoma (Alliance A091401): two open-label, non-comparative, randomised, phase 2 trials. Lancet Oncol. (2018) 19:416–26. doi: 10.1016/s1470-2045(18)30006-8 PMC6126546 29370992

[7] Chen AP Sharon E O’Sullivan-Coyne G Moore N Foster JC Hu JS. Atezolizumab for advanced alveolar soft part sarcoma. N Engl J Med. (2023) 389:911–21. doi: 10.1056/NEJMoa2303383 PMC10729808 37672694

[8] Panagi M Pilavaki P Constantinidou A Stylianopoulos T. Immunotherapy in soft tissue and bone sarcoma: unraveling the barriers to effectiveness. Theranostics. (2022) 12:6106–29. doi: 10.7150/thno.72800 PMC9475460 36168619

[9] Livingston MB Jagosky MH Robinson MM Ahrens WA Benbow JH Farhangfar CJ. Phase II study of pembrolizumab in combination with doxorubicin in metastatic and unresectable soft-tissue sarcoma. Clin Cancer Res. (2021) 27:6424–31. doi: 10.1158/1078-0432.Ccr-21-2001 34475102

[10] Haddox CL Nathenson MJ Mazzola E Lin JR Baginska J Nau A. Phase II study of eribulin plus pembrolizumab in metastatic soft-tissue sarcomas: clinical outcomes and biological correlates. Clin Cancer Res. (2024) 30:1281–92. doi: 10.1158/1078-0432.Ccr-23-2250 PMC10982640 38236580

[11] Lee AQ Hao C Pan M Ganjoo KN Bui NQ. Histologic and immunologic factors associated with response to immune checkpoint inhibitors in advanced sarcoma. Clin Cancer Res. (2025) 31:678–84. doi: 10.1158/1078-0432.CCR-24-3485 39699310

[12] Saerens M Brusselaers N Rottey S Decruyenaere A Creytens D Lapeire L. Immune checkpoint inhibitors in treatment of soft-tissue sarcoma: A systematic review and meta-analysis. Eur J Cancer. (2021) 152:165–82. doi: 10.1016/j.ejca.2021.04.034 34107450

[13] National Comprehensive Cancer Network. Soft tissue sarcoma (2025). Available online at: https://www.nccn.org/guidelines/guidelines-detail?category=1&id=1464 (Accessed February 15, 2025).

[14] Tawbi HA Burgess M Bolejack V Van Tine BA Schuetze SM Hu J. Pembrolizumab in advanced soft-tissue sarcoma and bone sarcoma (SARC028): a multicentre, two-cohort, single-arm, open-label, phase 2 trial. Lancet Oncol. (2017) 18:1493–501. doi: 10.1016/s1470-2045(17)30624-1 PMC7939029 28988646

[15] Wagner MJ Othus M Patel SP Ryan C Sangal A Powers B. Multicenter phase II trial (SWOG S1609, cohort 51) of ipilimumab and nivolumab in metastatic or unresectable angiosarcoma: a substudy of dual anti-CTLA-4 and anti-PD-1 blockade in rare tumors (DART). J Immunother Cancer. (2021) 9:e002990. doi: 10.1136/jitc-2021-002990 34380663 PMC8330584

[16] Hindi N Razak A Rosenbaum E Jonczak E Hamacher R Rutkowski P. Efficacy of immune checkpoint inhibitors in alveolar soft-part sarcoma: results from a retrospective worldwide registry. ESMO Open. (2023) 8:102045. doi: 10.1016/j.esmoop.2023.102045 38016251 PMC10698259

[17] Blumenthal GM Zhang L Zhang H Kazandjian D Khozin S Tang S. Milestone analyses of immune checkpoint inhibitors, targeted therapy, and conventional therapy in metastatic non-small cell lung cancer trials: a meta-analysis. JAMA Oncol. (2017) 3:e171029. doi: 10.1001/jamaoncol.2017.1029 28617920 PMC5824222

[18] Fujiwara T Nakata E Kunisada T Ozaki T Kawai A. Alveolar soft part sarcoma: progress toward improvement in survival? A population-based study. BMC cancer. (2022) 22:891. doi: 10.1186/s12885-022-09968-5 35971085 PMC9377116

[19] Dalton HJ Pradeep S McGuire M Hailemichael Y Ma S Lyons Y. Macrophages facilitate resistance to anti-VEGF therapy by altered VEGFR expression. Clin Cancer Res. (2017) 23:7034–46. doi: 10.1158/1078-0432.Ccr-17-0647 PMC5690831 28855350

[20] Meyer CF. Immunotherapy for sarcoma: a work in progress. J Clin Oncol. (2022) 40:1267–70. doi: 10.1200/jco.21.01338 35259000

[21] Baa AK Rastogi S. Combination doxorubicin and pembrolizumab in patients with advanced anthracycline-naive sarcoma. JAMA Oncol. (2021) 7:465. doi: 10.1001/jamaoncol.2020.7868 33507225

[22] Schöffski P Bahleda R Wagner AJ Burgess MA Junker N Chisamore M. Results of an Open-label, Phase Ia/b Study of Pembrolizumab plus olaratumab in patients with unresectable, locally advanced, or metastatic soft-tissue sarcoma. Clin Cancer Res. (2023) 29:3320–8. doi: 10.1158/1078-0432.Ccr-23-0742 PMC10472093 37382656

[23] Gordon EM Chawla SP Tellez WA Younesi E Thomas S Chua-Alcala VS. SAINT: A Phase I/expanded phase II study using safe amounts of ipilimumab, nivolumab and trabectedin as first-line treatment of advanced soft tissue sarcoma. Cancers. (2023) 15:906. doi: 10.3390/cancers15030906 36765863 PMC9913367

[24] ASCO. Home (2025). Available online at: https://www.asco.org/abstracts-presentations/ABSTRACT409688 (Accessed February 15, 2025).

[25] Tian Z Dong S Zuo W Li P Zhang F Gao S. Efficacy and safety of sintilimab plus doxorubicin in advanced soft tissue sarcoma: A single-arm, phase II trial. Front Pharmacol. (2022) 13:987569. doi: 10.3389/fphar.2022.987569 36582535 PMC9793899

[26] European Society for Medical Oncology. OncologyPRO (2025). Available online at: https://oncologypro.esmo.org/meeting-resources/esmo-congress-2022/phase-i-ii-study-to-evaluate-penpulimab-combined-with-anlotinib-and-epirubicin-in-the-first-line-treatment-of-soft-tissue-sarcoma-phase-i-dose-esc (Accessed February 15, 2025).

[27] Burtness B Harrington KJ Greil R Soulières D Tahara M de Castro. Pembrolizumab alone or with chemotherapy versus cetuximab with chemotherapy for recurrent or metastatic squamous cell carcinoma of the head and neck (KEYNOTE-048): a randomised, open-label, phase 3 study. Lancet. (2019) 394:1915–28. doi: 10.1016/s0140-6736(19)32591-7 31679945

